# Gartanin induces cell cycle arrest and autophagy and suppresses migration involving PI3K/Akt/mTOR and MAPK signalling pathway in human glioma cells

**DOI:** 10.1111/jcmm.12937

**Published:** 2016-08-05

**Authors:** Ming Luo, Qingyu Liu, Mingliang He, Zhiling Yu, Rongbiao Pi, Min Li, Xiaohong Yang, Shengnan Wang, Anmin Liu

**Affiliations:** ^1^ Guangdong Provincial Key Laboratory of Malignant Tumor Epigenetics and Gene RegulationSun Yat‐Sen Memorial HospitalSun Yat‐Sen UniversityGuangzhouChina; ^2^ Department of OncologySun Yat‐Sen Memorial HospitalSun Yat‐Sen UniversityGuangzhouChina; ^3^ Department of RadiologySun Yat‐Sen Memorial HospitalSun Yat‐Sen UniversityGuangzhouChina; ^4^ Department of NeurosurgerySun Yat‐Sen Memorial HospitalSun Yat‐Sen UniversityGuangzhouChina; ^5^ School of Chinese MedicineHong Kong Baptist UniversityHong KongChina; ^6^ School of Pharmaceutical SciencesSun Yat‐Sen UniversityGuangzhouChina; ^7^ International Joint Laboratory (SYSU‐PolyU HK) of Novel Anti‐dementia Drugs of GuangdongGuangzhouChina

**Keywords:** gartanin, proliferation, migration, malignant glioma

## Abstract

In central nervous system, glioma is the most common primary brain tumour. The diffuse migration and rapid proliferation are main obstacles for successful treatment. Gartanin, a natural xanthone of mangosteen, suppressed proliferation, migration and colony formation in a time‐ and concentration‐dependent manner in T98G glioma cells but not in mouse normal neuronal HT22 cells. Gartanin, at low micromole, led to cell cycle arrest in G1 phase accompanied by inhibited expression level of G1 cell cycle regulatory proteins cyclin D1, while increased expression level of cyclin‐dependent kinase inhibitor p27Kip1. In addition, the secretion and activity of matrix metalloproteinases 2/9 (MMP‐2/‐9) were significantly suppressed in T98G cells treated with gartanin, and it might result from modulating mitogen‐activated protein kinases (MAPK) signalling pathway in T98G glioma cells. Moreover, gartanin significantly induced autophagy in T98G cells and increased GFP‐LC3 punctate fluorescence accompanied by the increased expression level of Beclin 1 and LC3‐II, while suppressed expression level of p62. Gartanin treatment resulted in obvious inhibition of PI3K/Akt/mTOR signalling pathway, which is important in modulating autophagy. Notably, gartanin‐mediated anti‐viability was significantly abrogated by autophagy inhibitors including 3‐methyladenine (3‐MA) and chloroquine (CQ). These results indicate that anti‐proliferation effect of gartanin in T98G cells is most likely via cell cycle arrest modulated by autophagy, which is regulated by PI3K/Akt/mTOR signalling pathway, while anti‐migration effect is most likely via suppression of MMP‐2/‐9 activity which is involved in MAPK signalling pathway.

## Introduction

Glioma is aggressive and common primary tumour in central nervous system (CNS) 1. Their main characteristics are rapid proliferation and extensive migration. Surgery, radiation and chemotherapy are effective therapies for gliomas. Despite these treatments, the median survival time of glioblastoma patients is still about 15 months 2, 3. The effectiveness of glioma therapy was influenced by many factors including rapid tumour growth and highly infiltrative nature of glioma cells 4, 5, 6. Therefore, suppressing proliferation and inhibiting glioma cells migration would be a promising therapeutic strategy.

Mangosteen, *Garcinia mangostana* L., a common Southeast Asia tropical fruit, has been consumed as food and medicine for centuries 7. Xanthones are characterised by one or more hydroxy and prenyl groups in their tricyclic ring system. Cumulative evidence indicates that xanthones regulate diverse biologic processes such as antioxidation 8, anti‐tumour 9, anti‐inflammation 10, anti‐allergy 11, anti‐bacteria, anti‐fungi and anti‐virus 12. Recently, there has been reported that tumours could be suppressed by several kinds of xanthones isolated from the pericarp of mangosteen including gartanin 13, 14, α‐mangostin 15, 16 and γ‐mangostin 17, 18, and were recognised as potential anti‐cancer drugs. α‐Mangostin and γ‐mangostin have been extensively studied in a variety of neoplasm. By now, there was no report on the effects of gartanin on glioma development yet.

In this research, we found that gartanin, at lower micromole, potently inhibited the migration and viability abilities in T98G cells. Further studies showed that the anti‐tumour effects of gartanin might involve cell cycle arrest in G1, increased protein expression level of p27^Kip1^, suppressed protein expression level of cyclin D1 and inhibited secretion and activity of MMP‐2/‐9. Moreover, the anti‐viability effect of gartanin was also associated with autophagy. Further studies indicated that PI3K/Akt/mTOR was associated with gartanin‐induced autophagy and mitogen‐activated protein kinases (MAPK) signalling pathways were involved in the suppressed expression level and activity of MMP‐2/‐9. In summary, results indicate that gartanin might be a promising anti‐tumour drug against gliomas.

## Materials and methods

### Antibodies and reagents

Gartanin, γ‐mangostin, garciniafuran, garcinone C, 8‐deoxygartanin, α‐mangostin and garcinone D isolated from the fruit hulls of mangosteen were kindly provided by Professor Rongbiao Pi (Zhongshan University) and their purity was tested to be over 99% *via* high‐performance liquid chromatography (HPLC). Antibodies against cyclin D1, p27^Kip1^, p‐Erk (thr202/tyr204), p‐JNK (thr183/tyr185), p‐p38 (thr180/tyr182), p‐Akt (ser473), Akt, Erk, p‐GSK‐3β (ser9), LC3, Beclin 1, p62, GAPDH, α‐tubulin and β‐actin were purchased from Sigma‐Aldrich (St. Louis, MO, USA).

### Cell culture

U87, U251, T98G human malignant glioma cells and HT22 murine hippocampal neuronal cells were kindly provided by Professor Rongbiao Pi (Zhongshan University). Cells mentioned above were maintained in DMEM (Hyclone, Grand Island, NY, USA) supplemented with 10% FBS (Gibco, Grand Island, NY, USA), 100 μg/ml streptomycin and 100 units/ml penicillin (Sigma, USA). Cells were maintained in an incubator with 5% CO_2_. Gartanin, γ‐mangostin, garciniafuran, α‐mangostin, 8‐deoxygartanin, garcinone D and garcinone C were dissolved in DMSO.

### Cell viability and colony formation assays

MTT assay was used to test cell viability and lactate dehydrogenase (LDH) assay was used to evaluate cytotoxicity. Briefly, cells were planted in 96‐well plates. After 50% confluence was reached, cells were treated with gartanin at various concentrations for various time spans, and then MTT (10 μl) was added into every well after that maintained in the incubator for 2 hr. Finally, DMSO (100 μl) was added into every well after the removal of MTT solution. A microplate reader (Bio‐Tek, Winooski, VT, USA) was used to test the value of optical density (OD) at 570 nm. As for colony formation, cells at a density of 60 cells/well were planted in six‐well plates. After cultured in incubator for 7 days, cells were fixed with 4% paraformaldehyde solution and then dyed with 1.0% crystal violet. An inverted microscope (XDS‐1B, COIC, Chongqing, China) was used to count the number of colonies. LDH release in the supernatant was determined with a cytotoxicity assay kit (Shenggong, China) according to the manufacturer's instructions. A microplate reader (Bio‐Tek) was used to test the value of OD at 490 nm.

### Apoptosis cells staining

Cells were planted in 96‐well plates. After 50% confluence was reached, cells were treated with gartanin at various concentrations for various time spans. After treatment, cells were fixed with 4% paraformaldehyde solution for half an hour and then dyed with DAPI for another half an hour and photographed with a fluorescence microscope (IX71, Chongqing, China). Apoptotic cells were characterised by morphological alternation as condensed nuclei and cell shrinkage.

### Cell cycle analysis

The cell cycle phase distribution was determined *via* flow cytometry analysis of DNA content of cells with a cell cycle assay kit (Nanjing, China) according to the instructions. In brief, T98G was planted in six‐well plates and treated with gartanin at various concentrations for the indicated time. After treatment, cells were trypsinised and fixed with 65% ethanol for 12 hrs. Cells were then incubated with DNA‐binding dye propidium iodide (PI, 50 μg/ml) and RNase (1.0 mg/ml) for another 1 hr in the incubator. Finally, a flow cytometry (Beckman, Heidelberg, Germany) was used to analyse the red fluorescence, and a peak fluorescence gate was used to discriminate aggregates.

### Wound healing assay

T98G cells were planted in six‐well plates and cultured in incubator. When the cells reached confluence, would healing assay was performed. In brief, a 200‐μl pipette tip was used to manually scrape the cell monolayer. The floating cells were washed away with PBS. Cells were then maintained in DMEM. Cell migration was observed at three indicated time points (0, 12 and 24 hrs) in three different microscopic fields for each concentration of gartanin. Images were acquired with a phase‐contrast microscope (IX71, Chongqing, China) and were processed using Adobe Photoshop 7.0. The wound width at time 0 minus the wound width at different time points is the distance migrated by the cells. The values were expressed as a migration percentage, and setting the 0 hr group as 0%.

### Real‐time cell analysis assay: migration

XCELLigence real‐time cell analysis (RTCA) DP system (Roche, Mannheim, Germany) was used to perform cell migration experiments. Real‐time cell analysis was allowed by the xCELLigence system on the RTCA DP Instrument equipped with a CIM‐Plate 16. In brief, T98G cells were trypsinised and resuspended in DMEM. Each upper chamber was planted with 3 × 10^4^ cells to allow cells attachment, the CIM‐Plate was maintained in incubator for another 30 min. The xCELLigence system automatically monitored the impedance value of each well for duration of 24 hrs, and a CI value was obtained.

### Gelatin zymography assay

A gelatin zymography assay kit (Applygen, Beijing, China) was used to measure the activity of MMP‐2 and MMP‐9 in the supernatant. In brief, cells were planted in six‐well plates and treated with gartanin at various concentrations for 24 hrs. Ten microlitre supernatant of each well was collected and was mixed with 10 μl sample buffer. An equal volume of sample (20 μl) was then loaded in each lane of 8% SDS‐PAGE containing gelatin as a substrate. Using pre‐cooled electrophoresis running buffer, electrophoresis was carried out at 20 mA for 60 min. After washing with 2% Triton X‐100, gels were dipped into the developing buffer containing 0.02% NaN_3_, 50 mM Tris and 10 mM CaCl_2_ for 42 hrs at 37°C. R‐250 of Coomassie brilliant blue was used to dye the gels, and 25% methanol mixed with 10% glacial acetic acid was used to destain the gels. After this, the bands were visualised. A transparent band on a blue background indicates the proteolytic activity of each sample. Image J software (NIH, Bethesda, MD, USA), version 1.46r, was used to perform quantitative analysis.

### GFP‐LC3 transient transfection

Plasmid, GFP‐LC3, was kindly provided by Professor Rongbiao Pi (Sun Yat‐Sen University). T98G cells (2 × 10^5^ cells/well) were planted on a glass coverslip in the six‐well plates and cultured in incubator. After reaching 60% confluence, Lipofectamine 3000 transfection reagent (Invitrogen, Carlsbad, California, USA) was used to perform transient transfection in accordance with the instructions. After 8 hrs, the supernatant was removed and complete medium was added. After the sample was incubated for 1 day, cells were treated with gartanin at various concentrations for another 7 hrs after which it was fixed with 4% paraformaldehyde solution for half an hour at 37°C. Confocal laser microscopy (Zeiss LSM710, Jena, Germany) was used to analyse the cells.

### Western blot

After treatment, cells were collected, washed and then lysed in 100 μl lysis buffer (10 mM NaF, protease inhibitor cocktail, 20 mM Tris‐HCl, 150 mM NaCl, 1 mM EDTA, 2 mM Na_3_VO_4_ and 1% Triton X‐100, pH 7.5). BCA assay kit (Thermofisher Pierce, Rockford, AL, USA) was used to determine the protein concentration. An equal amount of protein (20 μg) was loaded into lanes. After separated by electrophoresis, proteins were electrically transferred to a PVDF membrane (Millipore, Billerica, MA, USA). After the membrane blockage, specific antibodies were used to immunodetect the target proteins. After incubated with corresponding secondary antibody, target proteins were developed by the enhanced chemiluminescence technique.

### Statistical analysis

The experimental data were presented as mean ± S.D. Differences among groups were analysed by one‐way analysis of variance (anova). Following anova analyses, the Tukey's test was used. Differences between two groups were analysed by unpaired Student's *t*‐test. Experiments described in this study were repeated at least three times. *P <* 0.05 was considered to be statistically significant.

## Results

### Anti‐viability effect of gartanin

**Figure 1:**
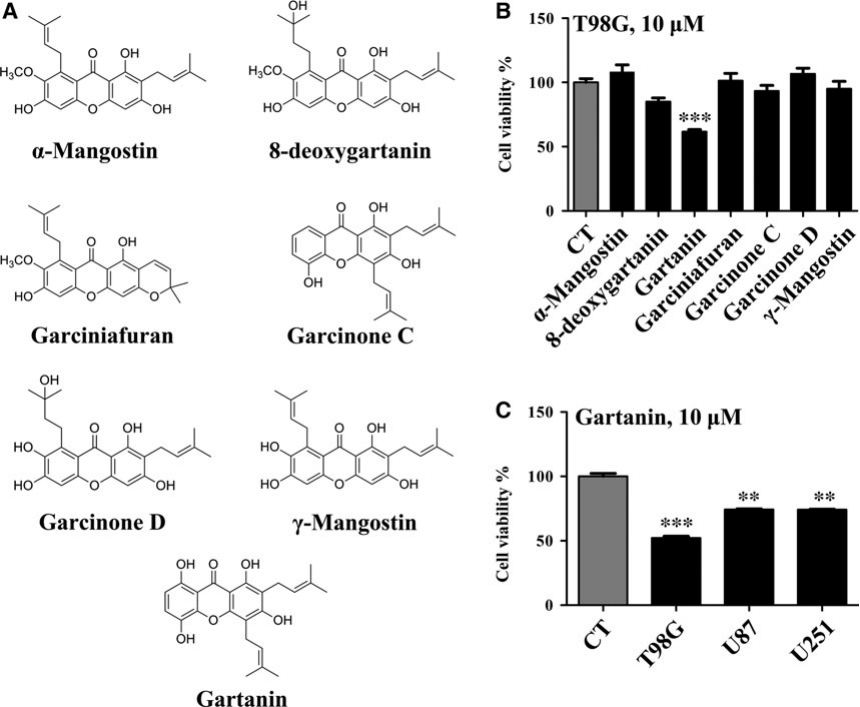
Gartanin specifically suppressed the viability of glioma cells. (**A**) Chemical structure of seven *Garcinia mangostana* extracts. (**B**) The inhibitory effects of seven *G. mangostana* extracts on T98G cells analysed by MTT assay. (**C**) The inhibitory effects of gartanin on T98G, U87 and U251 malignant glioma cells analysed by MTT assay. **P < 0.01, ***P < 0.001 *versus* the control group.

In our preliminary experiments, we tested the anti‐viability effects of seven xanthones isolated from the shells of *Garcinia mangostana* L. (Fig. 1A) and found that the potency of gartanin was by far the greatest in T98G cells (Fig. 1B). Thus, all subsequent experiments were focused on gartanin. As shown in Fig. 1C, three malignant glioma cell lines including T98G, U87 and U251 were all sensitive to the anti‐viability effect of gartanin, and T98G was the most sensitive one. So, we did the subsequent experiments in T98G cells to uncover the characters of gartanin's anti‐cancer activity as well as the underlying mechanisms.

### Gartanin specifically inhibits cell viability and colony formation of T98G glioma cells, but does not induce cell death

**Figure 2:**
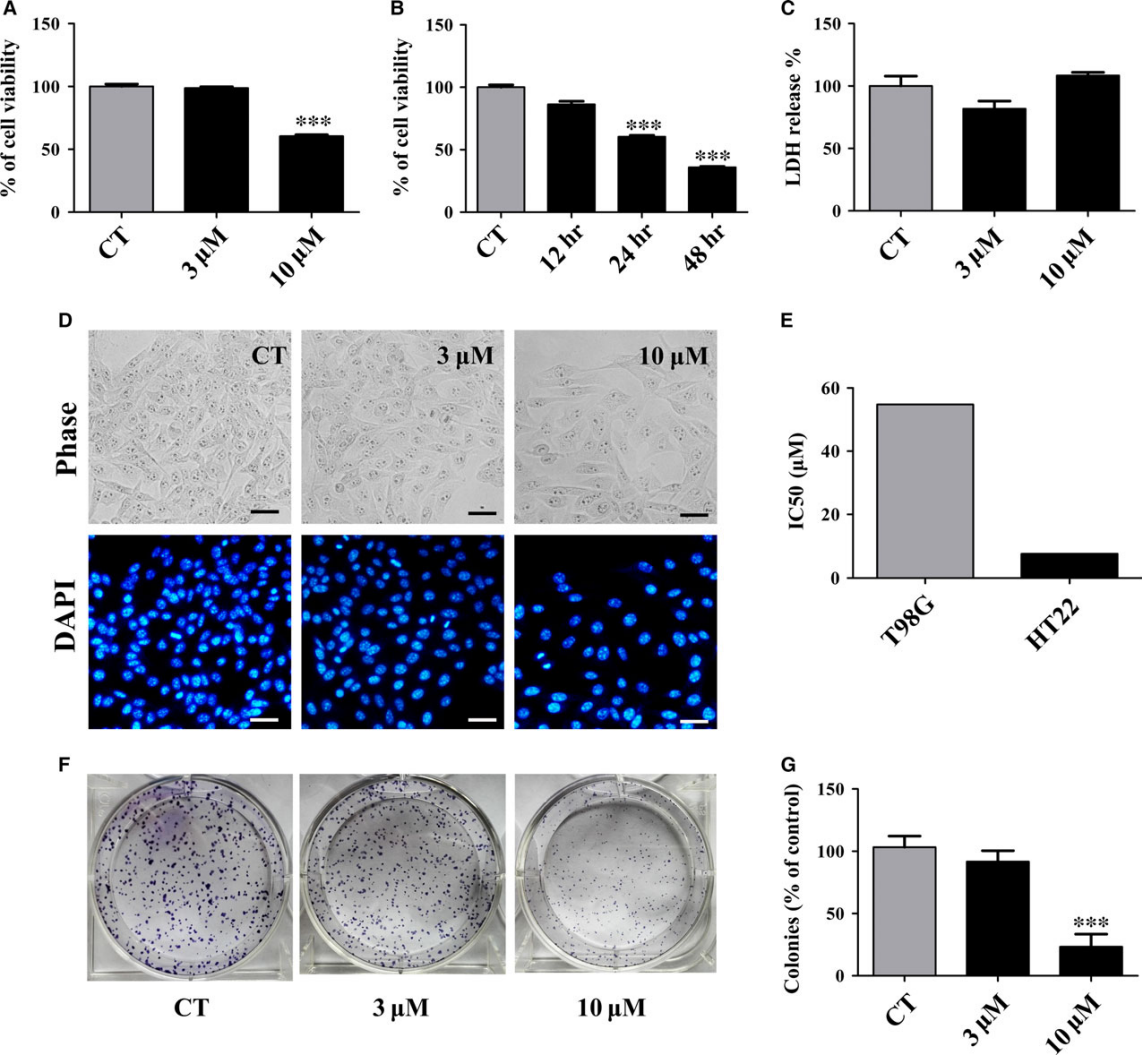
Gartanin induces viability inhibition but not cell death, and inhibits colony formation in T98G glioma cells. (**A/B**) T98G cells were treated with Gartanin at indicated concentrations and periods of time. MTT absorbance was measured for cell viability. ***P < 0.001 compared with the control group. (**C**) LDH absorbance was measured for cytotoxicity. (**D**) DAPI staining of T98G cell nuclei treated with 3 μM or 10 μM gartanin. The cell morphology was observed under a microscope (200×). Scale bar = 20 μm. (**E**) The IC50 of gartanin for indicated cell lines. (**F**) T98G cells were treated with gartanin at indicated concentrations. Colony formation ability is measured. One of three independent experiments is shown. (**G**) Statistical analysis of three independent experiments. ***P < 0.001 *versus* the control group.

Active mitochondria within cells are required in the process of conversing MTT to a blue formazan. Thus, cellular activity could be measured by MTT scores. Figure 2A and B indicated that gartanin suppressed the cell viability of T98G cells in a dose‐ and time‐dependent manner. A 46.5% inhibition rate (*P* < 0.001, *n* = 3) was observed in cells treated with gartanin (10 μM) for 24 hrs. The IC_50_ of gartanin on the growth of T98G was 10.8 μM. However, the IC_50_ on that of normal mouse neuron HT22 cells was 54.2 μM (Fig. 2E).

Either cell death or reduced viability could result in the reduction of MTT score. To determine whether gartanin caused necrosis or apoptosis, LDH assay and DAPI staining were used. No significant difference was observed in the number of apoptotic bodies stained with DAPI between gartanin‐treated group and control group (Fig. 2D). No obvious difference was observed in LDH release between gartanin‐treated group and control group (Fig. 2C). Therefore, reduction in MTT score was not resulted from cytotoxicity. Thus, significantly reduced MTT score in gartanin‐treated group most likely resulted from decreased viability. Consistently, colony formation assay showed that, compared with control group, cells treated with gartanin (10 μM) formed significantly smaller and fewer colonies (Fig. 2F and G; ***P < 0.001).

### Gartanin arrests cell cycle progression in T98G cells

**Figure 3:**
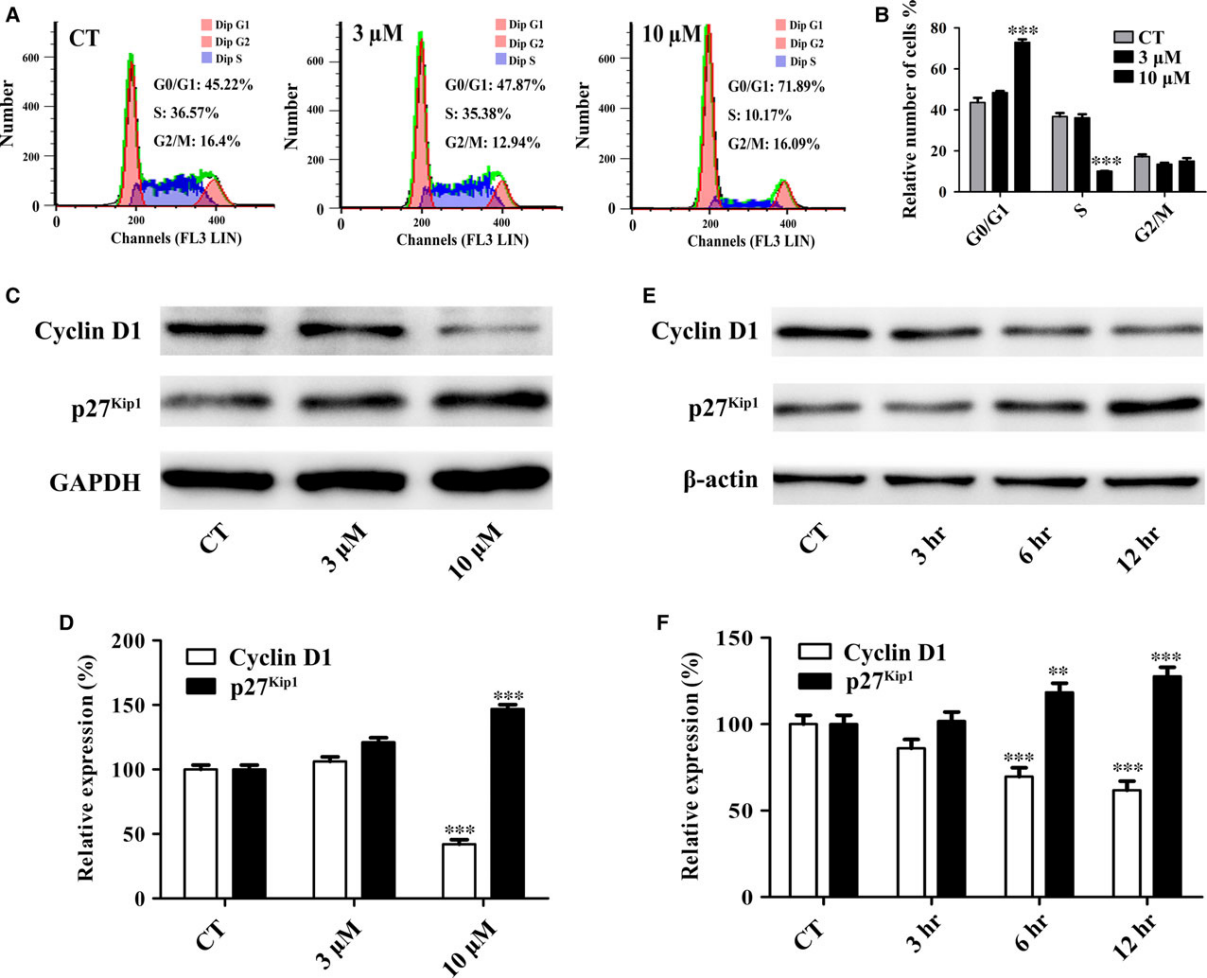
Gartanin` induces G1 phase cell cycle arrest accompanied by suppressed G1 cell cycle regulatory proteins of T98G glioma cells. (**A**) Cell cycle distributions. One of three independent experiments is shown. (**B**) Statistical analysis of three independent experiments. ***P < 0.001 *versus* the control group. (**C/E**) T98G cells were treated with gartanin at indicated concentrations and periods of time. Western blots analysis of cyclin D1 and p27^Kip1^. (**D/F**) Relative density of cyclin D1 and p27^Kip1^ is determined by densitometry of the blots. **P < 0.01, ***P < 0.001 *versus* the control group.

To preliminarily reveal the mechanism of viability suppression in gartanin‐treated group, flow cytometry was used to determine cell cycle distribution in T98G cells. Results showed that gartanin led to G1 phase accumulation in a dose‐dependent manner to reach 47.87% and 71.89% of gartanin at 3 and 10 μM, respectively, while 45.22% was observed in control group. Concomitantly, gartanin decreased the cells in S fraction from 36.57% of control group to 10.17% of gartanin (10 μM) group (*P* < 0.001). Moreover, the change in the G2/M phase did not reach statistical significance (Fig. 3A and B). These results indicate that gartanin caused cell viability suppression in T98G cells possibly because of G0/G1 cell cycle arrest.

To explore the molecular basis of the cell cycle arrest in gartanin‐treated group, Western blot was used to investigate the expression of some cell cycle regulatory proteins. As the checkpoint of G1/S phase, the expression of cyclin D1 was investigated. Figure 3C and D showed that the protein expression level of cyclin D1 was significantly reduced in gartanin‐treated group in a time‐ and concentration‐dependent manner. Additionally, as an important negative regulatory factor of cell cycle, the expression level of cyclin‐dependent kinase (CDK) inhibitor, p27^Kip1^, was investigated. Figure 3E and F indicated that the protein expression of p27^Kip1^ was significantly increased in gartanin‐treated group in a time‐ and concentration‐dependent manner. Data indicated that inactivation of cyclin D1 accompanied by activation of p27^Kip1^ attributed to arrest cell cycle progression from G1 to S phase, further resulted in viability suppression in gartanin‐treated group.

### Gartanin suppresses T98G glioma cells migration

**Figure 4:**
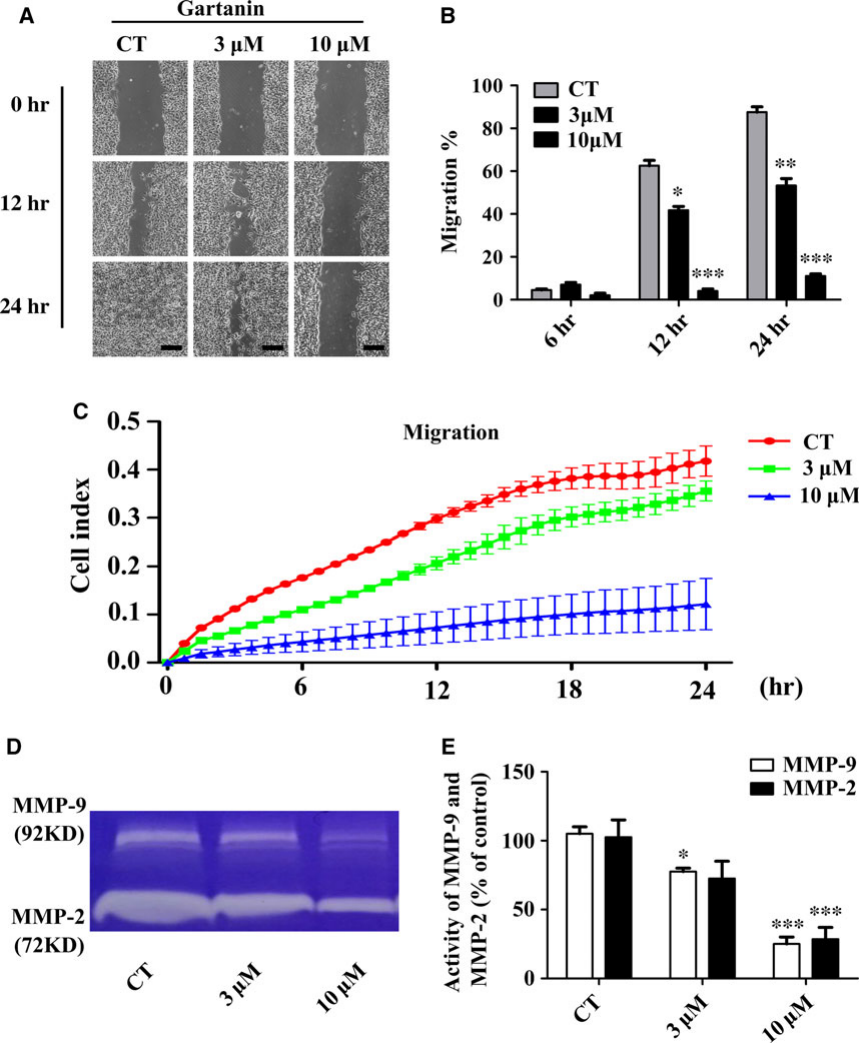
Gartanin inhibits migration ability and proteolytic activities of MMP‐2/‐9 in T98G cells. (**A**) Cell migration ability was measured by wound healing assay in T98G cells. Representatives of three independent experiments are shown (50×). Scale bar = 100 μm. (**B**) Quantification of cell motility by measuring the wound width. *P < 0.05, **P < 0.01, ***P < 0.001 *versus* the control group. (**C**) Migration capacity of T98G cells in various gartanin concentrations are continuously monitored by real‐time cell analysis for 24 hrs and plotted by cell index. (**D**) MMP‐2/‐9 proteolytic activities are determined by gelatin zymography in supernatant collected 24 hrs after treatment with gartanin. An active MMP‐2 protein (72 kDa) and MMP‐9 (92 kDa) are shown. (**E**) Statistical analysis of MMP‐2/‐9 proteolytic activities of three independent experiments. ***P < 0.001 *versus* the control group.

Malignant gliomas are not only characterised by infinite proliferation ability but also by highly migration ability. Wound healing assay was performed to determine the possible effects of gartanin on migration. Figure 4A showed that the scratched area was completely re‐colonised after 24 hrs in the control group. However, this process was significantly impaired in a dose‐dependent manner in gartanin‐treated group. As a matter of fact, cells treated with 10 μM gartanin rarely invaded the scratched area. Moreover, there was statistically significant difference in the migration distance between treated group and the control group 12 hrs after scratch (Fig. 4A). Quantitative analysis of wound closure clearly showed that 10 μM gartanin group closed 10.1% and 15.2% of the wound after 12 and 24 hrs respectively. Conversely, the control group closed 63.2% and 96.3% of the wound after 12 and 24 hrs respectively (Fig. 4B). Results indicated that migration of T98G malignant glioma cells could be significantly suppressed by gartanin. To eliminate interference factors including invasion and proliferation, RTCA assay was performed. The migration of T98G cells from the upper chambers to the lower chamber was monitored by the electrodes. Data indicated that T98G cells treated with 10 μM gartanin migrated significantly slower when compared with the controls (Fig. 4C).

To explore the molecular basis of anti‐migration in gartanin‐treated group, the activities of MMP‐2 and MMP‐9 were determined by gelatin zymography assay. Figure 4D indicated that the activities of MMP‐9 and MMP‐2 were significantly suppressed by gartanin. As shown in Figure 4E, quantitative analysis suggested that MMP‐2 activity reduced by 27.5% and 71.5% and MMP‐9 activity reduced by 22.5% and 75.0% when cells were treated with 3 μM or 10 μM gartanin respectively.

### MAPK signalling pathway involving the proteolytic activities of MMP‐2/‐9 was suppressed by gartanin

**Figure 5:**
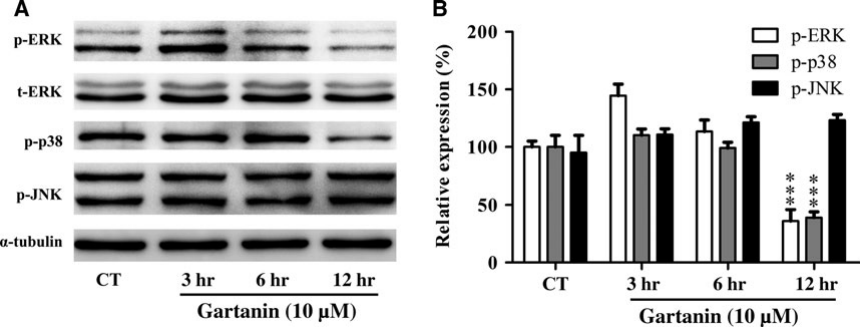
Gartanin suppresses ERK, p38 MAPK in T98G glioma cells. (**A**) T98G cells were treated with 10 μM of gartanin for indicated periods of time. The phosphorylated and total protein levels of ERK, JNK and p38 MAPK are assayed by Western blot. Representatives of three independent experiments are shown. (**B**) Relative density of p‐ERK, p‐JNK and p‐p38 are determined by densitometry of the blots. ***P < 0.001 *versus* the control group. MAPK, mitogen‐activated protein kinases.

To gain insight into the signalling pathway involving the modulation of MMP‐9 and MMP‐2, we assessed the effects of gartanin on MAPK signalling pathway. Specifically, the activation of several different downstream MAPK including p‐ERK1/2 (thr202/tyr204), p‐JNK (thr183/tyr188) and p‐p38 (thr180/tyr182) were assessed. Figure 5 showed that gartanin (10 μM) time dependently suppressed the expression level of p‐p38 and p‐ERK. And the expression level of total ERK did not change. Moreover, no changes in the phosphorylated forms of JNK were observed.

### Gartanin induces T98G glioma cells autophagy

**Figure 6:**
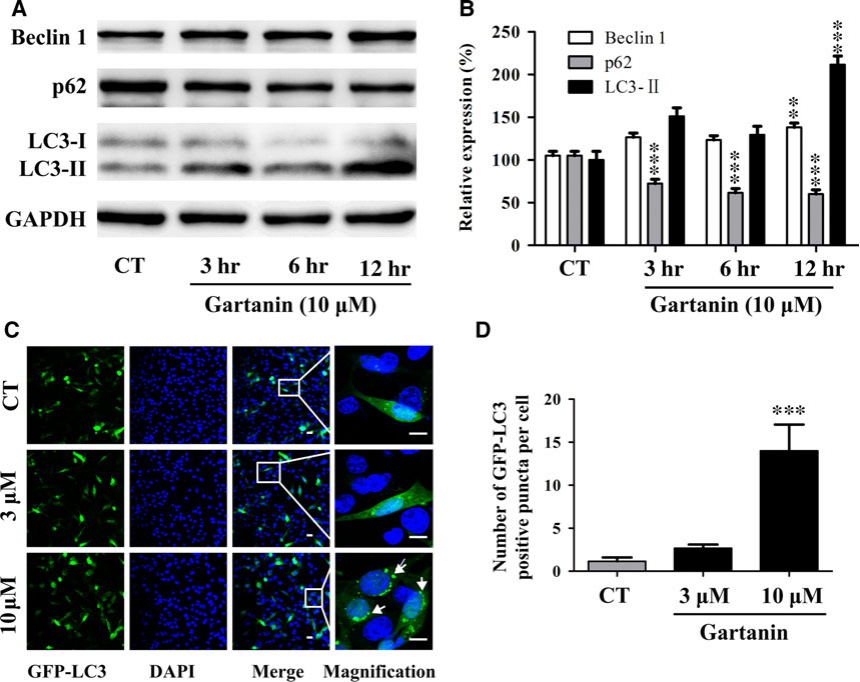
Gartanin induces autophagy in T98G glioma cells. (**A**) T98G cells are treated with 10 μM of gartanin for indicated periods of time. The protein levels of Beclin 1, p62 and LC3‐I/II are assayed by Western blot. Representatives of three independent experiments are shown. (**B**) Relative density of Beclin 1, p62 and LC3‐I/II are determined by densitometry of the blots. **P < 0.01, ***P < 0.001 *versus* the control group. (**C**) Representative images (200×) and higher magnification images (600×) of the indicated regions of T98G cells with green fluorescent protein‐light chain 3 (GFP‐LC3) puncta are shown. Scale bar = 100 μM. (**D**) Quantitative analysis of GFP‐LC3 positive puncta per cell. ***P < 0.001 *versus* the control group.

To determine whether autophagy was induced in glioma cells treated with gartanin, the protein expression levels of p62, LC3‐II and Beclin 1, which are important regulatory factors of autophagy, were detected by Western blot. Figure 6A and B showed that gartanin significantly increased the expression levels of Beclin 1 and LC3‐II, while significantly suppressed the expression level of p62 in a time‐dependent manner. Moreover, punctate structure, representing autophagosomes, could visualise the formation of LC3‐II. Thus, after transiently transfected with GFP‐LC3, cells were treated with gartanin at various concentrations for 8 hrs. Analysis was performed by confocal microscopy (Fig. 6C). Figure 6D showed that, compared with the control group (2.7%) and gartanin (3 μM) group (3.2%), the number of GFP‐LC3‐positive puncta per cell in gartanin (10 μM)‐treated group (16.5%) was significantly higher.

### PI3K/Akt/mTOR signalling pathway involving autophagy was suppressed by gartanin

**Figure 7:**
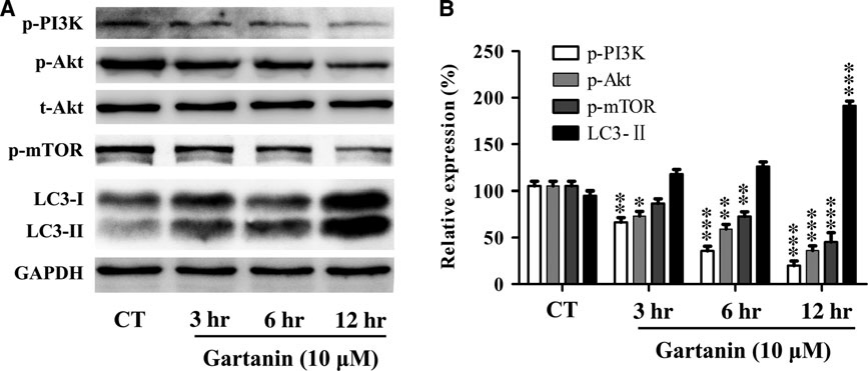
Gartanin inhibits PI3K/Akt/mTOR signalling pathway in T98G glioma cells. (**A**) T98G cells are treated with 10 μM of gartanin for indicated periods of time. The phosphorylated and total protein levels of PI3K, Akt, mTOR and LC3‐I/II are assayed by Western blot. Representatives of three independent experiments are shown. (**B**) Relative density of PI3K, Akt, mTOR and LC3‐I/II are determined by densitometry of the blots. *P < 0.05, **P < 0.01, ***P < 0.001 *versus* the control group.

To gain insight into the signalling pathway involving the regulation of autophagy in T98G malignant glioma cells, we assessed the effects of gartanin on PI3K/Akt/mTOR signalling pathway which was involved in inducing autophagy. Specifically, we detected three key proteins, p‐mTOR (ser2448), p‐PI3K (tyr458) and p‐Akt (ser473). As shown in Figure 7, gartanin (10 μM) time dependently suppressed the expression levels of p‐PI3K, p‐Akt, p‐mTOR and LC3‐II, while no changes were found in the content of total Akt.

### The anti‐viability effect of gartanin could be abrogated by autophagic inhibitors

**Figure 8:**
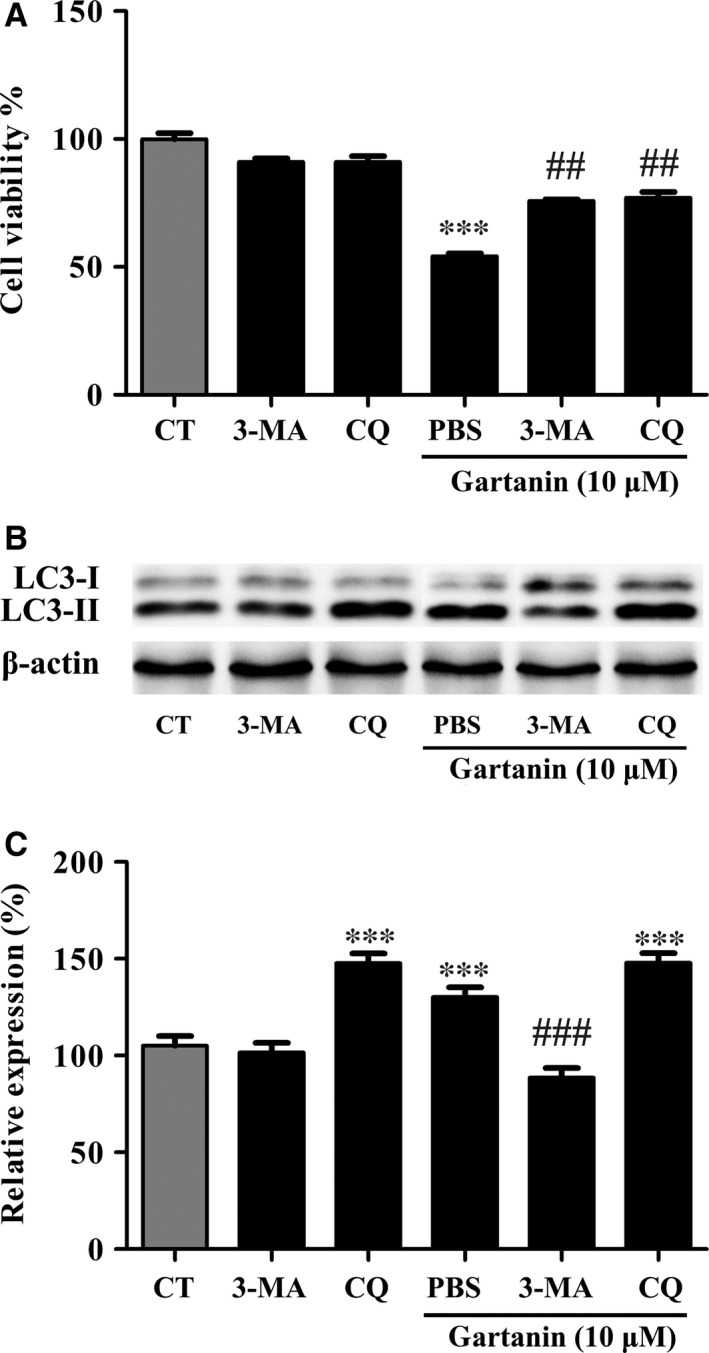
Autophagic inhibitors abolish the anti‐viability effect of gartanin in T98G glioma cells. (**A**) T98G cells are treated with 10 μM of gartanin with or without 3‐MA or CQ for 24 hrs, and the cell viability is detected by MTT assay. ***P < 0.001 *versus* the control group, ^##^ *P* < 0.01 *versus* the group of gartanin. (**B**) Cells are pre‐treated with the autophagy inhibitors 3‐MA and CQ, and then treated with 10 μM of gartanin for 24 hrs. Levels of LC3‐I/II are shown. (**C**) Relative density of LC3‐II is determined by densitometry of the blots. ***P < 0.001 *versus* the control group, ^###^ *P* < 0.001 *versus* the 10 μM gartanin group. CQ, chloroquine.

We tested the cell viability with or without autophagy inhibitors, chloroquine (CQ) or 3‐MA, to assess the effects of autophagy on the anti‐viability of gartanin. In brief, after pre‐treated with CQ (8 μM) or 3‐MA (2 mM), cells were treated with 10 μM gartanin. Western blot was used to investigate the expression level of LC3‐I/II, and MTT assay was used to investigate the cell viability. Figure 8B and C showed that expression level of LC3‐II was decreased by 3‐MA, while increased by CQ. Figure 8A showed that the cell viability of T98G was increased from 54% to 75.8% and 77%, respectively, by pre‐treatment with 3‐MA or CQ.

**Figure 9:**
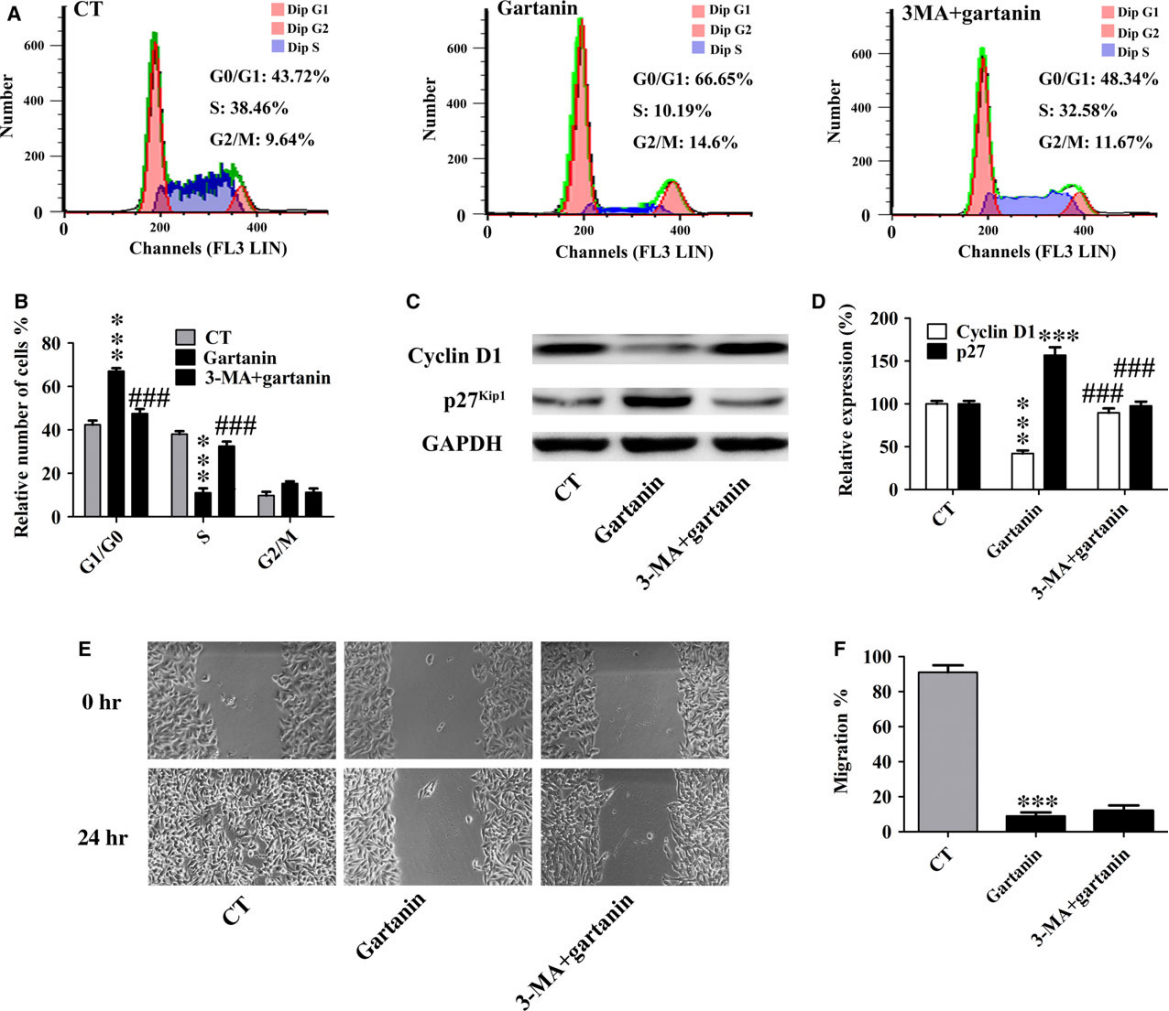
Autophagic inhibitor abrogated the arrest of cell cycle in G1 phase of gartanin in T98G glioma cells. (**A**) Cell cycle distributions. One of three independent experiments is shown. (**B**) Statistical analysis of three independent experiments. ***P < 0.001 *versus* the control group, ^###^ *P* < 0.001 *versus* the 10 μM gartanin group. (**C**) Western blot analysis of cyclin D1 and p27^Kip1^. (**D**) Relative density of cyclin D1 and p27^Kip1^ is determined by densitometry of the blots. ***P < 0.001 *versus* the control group, ^###^ *P* < 0.001 *versus* the 10 μM gartanin group. (**E**) Cell migration ability was measured by wound healing assay in T98G cells. Representatives of three independent experiments are shown (50×). Scale bar = 100 μm. (**F**) Quantification of cell motility by measuring the wound width. ***P < 0.001 *versus* the control group.

Further study showed that cells in G0/G1 phase decreased from 66.65% of 10 μM gartanin group to 48.34% of 3‐MA+10 μM gartanin group (*P* < 0.001), and cells in S phase increased from 10.19% of 10 μM gartanin group to 32.58% of 3‐MA+10 μM gartanin group (*P* < 0.001), although the change in the G2/M phase did not reach statistical significance (Fig. 9A and B). Regulatory proteins of G1 phase including cyclin D1 and p27^Kip1^ were investigated. Figure 9C and D showed that the decrease in cyclin D1 and increase in p27^Kip1^ were abolished by pre‐treatment with 3‐MA.

However, the anti‐migration effect of gartanin could not be abrogated by autophagic inhibitor, 3‐MA. Figure 9E and F showed that there was no significant restore of cell migration by pre‐treatment with 3‐MA.

### The anti‐viability effect of gartanin could not be abrogated by GSK‐3β inhibitor

**Figure 10:**
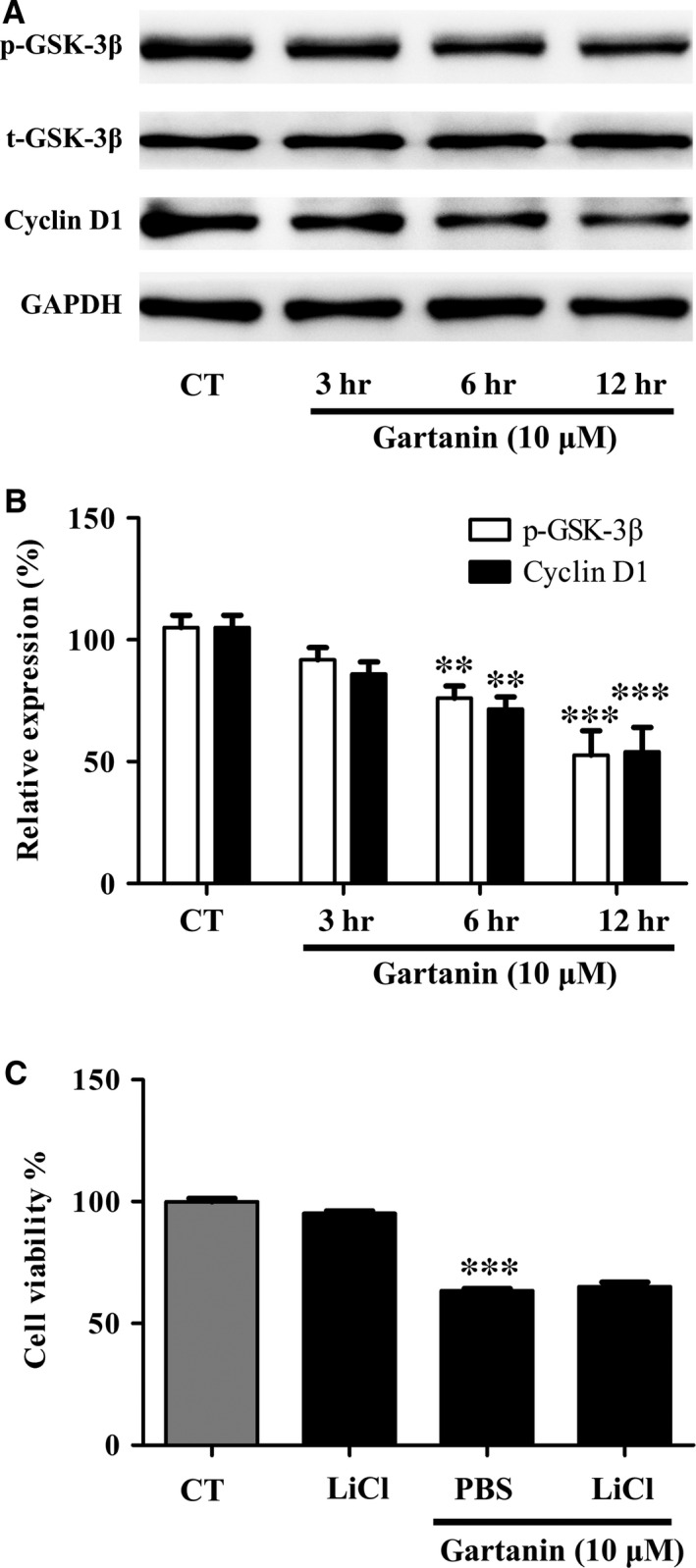
GSK‐3β inhibitors cannot abolish the anti‐viability effect of gartanin in T98G glioma cells. (**A**) Cells are pre‐treated with GSK‐3β inhibitors LiCl (10 mM), then treated with 10 μM of gartanin for 12 hrs. Levels of p‐GSK‐3β and cyclin D1 are shown. (**B**) Relative density of p‐GSK‐3β and cyclin D1 are determined by densitometry of the blots. **P < 0.01, ***P < 0.001 *versus* the control group. (**C**) T98G cells are treated with 10 μM of gartanin with or without LiCl for 24 hrs, and the cell viability is detected by MTT assay. ***P < 0.001 *versus* the control group.

We tested the cell viability with or without GSK‐3β inhibitor, LiCl, to assess the effects of GSK‐3β signalling pathway on the anti‐viability of gartanin. In brief, after pre‐treated with LiCl (0.5 mM), cells were treated with 10 μM gartanin. Western blot was used to investigate the expression level of GSK‐3β, and MTT assay was used to investigate the cell viability. Figure 10A and B showed that gartanin suppressed the expression levels of p‐GSK‐3β in a time‐dependent manner. Figure 10C showed that there was no significant restore of cell viability by pre‐treatment with LiCl.

## Discussion

Malignant gliomas are characterised by local migration and aggressive proliferation 19. Cell migration takes an important role in tumour invasiveness, metastasis and spreading. The infinite proliferative activity and infiltration nature of tumour cells are important factors in preventing the intact tumour resection, and thus resulting in tumour recurrence and therapeutic failure 20. In this study, results showed that gartanin could potently suppress migration and viability of T98G glioma cells (Fig. 1). It was worth to note that gartanin, at such concentrations, did not cause significant cytotoxicity in mouse normal neurons HT22 cells (Fig. 2E), indicating that gartanin might be a tumour‐specific agent. Besides, gartanin also has mighty neuroprotective effect against glutamate‐induced cell death in HT22 cells (data not shown and will be reported in another paper of our laboratory).

A fundamental reason for aberrant proliferation of tumour cells is dysregulation of cell cycle progression 21. In process of cell cycle, the first critical restriction point is the G1/S phase checkpoint. Cycle regulatory proteins, such as cyclins, CDKs and cyclin CDK inhibitors, play a key role in the G1/S transition 22. In particular, the cyclin D1 and p27^Kip1^ are essential for the regulation of cells to enter S phase. Previous studies showed that xanthones could induce G1 phase arrest in prostate cancer, lung cancer, breast cancer and hepatoma 23, 24, 25, 26. Consistently, this study showed that gartanin‐induced T98G cell G1 phase arrest accompanied by S phase proportion decreased (Fig. 3A and B). We also found that gartanin increased the expression level of p27^Kip1^, while decreased the expression level of cyclin D1 (Fig. 3C–F). Thus, anti‐viability effect of gartanin could be preliminary explained by cell cycle arrest because of the down‐regulation of positive cell cycle regulator and up‐regulation of negative cell cycle regulator. In addition, there has been reported that gambogenic acid, a polyprenylated xanthone, induced degradation of cyclin D1 *via* triggering dephosphorylation of GSK‐3β which is required for cyclin D1 turnover 23, 27. We have also found that gartanin suppressed the expression levels of p‐GSK‐3β. However, inactivation of GSK‐3β by LiCl cannot abrogate the anti‐viability effect of gartanin (Fig. 10). Therefore, the anti‐viability effect of gartanin might be irrelevant to GSK‐3β signalling pathway.

Besides GSK‐3β signalling pathway, proliferation of cancer is closely related to autophagy 28, 29. Autophagy, degrading cellular macromolecules or organelles, is an evolutionarily highly conserved catabolic pathway 30. The capacity of autophagy to maintain cellular metabolism improves the survival of cells. Paradoxically, autophagy has also been implicated in cell death called autophagic or type II programmed cell death 30. Previous studies have showed that xanthones, including α‐mangostin, gambogenic acid and gartanin, could induce protective autophagy or autophagic cell death in chronic myeloid leukaemia cells 23, human urinary bladder cancer cells 14, lung cancer cells 31, hepatocellular carcinoma cells 13 and glioblastoma cells 29. In this research, we also found that gartanin, a polyprenylated xanthone, obviously induced autophagy that was determined by the conversion of LC3‐I to LC3‐II, the degradation of p62, the increase in Beclin 1 and GFP‐LC3 punctate fluorescence (Fig. 6). Moreover, anti‐viability effect of gartanin was significantly abolished by CQ or 3‐MA, two autophagy inhibitors (Fig. 8). Further study showed that accumulation in G1 phase of gartanin group was significantly abrogated by pre‐treatment with 3‐MA (Fig. 9). Concomitantly, the decrease in cyclin D1 and increase in p27^Kip1^ were abolished by pre‐treatment with 3‐MA (Fig. 9). Accordingly, to some degree, the anti‐viability effect of gartanin on T98G cells involves cell cycle arrest which was regulated by autophagy. It is well known that PI3K/Akt/mTOR signalling pathway has been frequently implicated in regulating autophagy 32. Gartanin, here, inhibited key points of PI3K pathway, such as p‐PI3K, p‐Akt and p‐mTOR, in a time‐dependent manner (Fig. 7). Data suggest gartanin is a potentially effective anti‐viability agent against glioma, and this anti‐viability effect involves autophagy which is induced by inhibition of PI3K/Akt/mTOR pathway.

Local migration is another malignant phenotype of glioma. Although distant metastasis is pretty rare, the characteristic of the glioma cells that migrating into adjacent brain tissue makes intact resection impossible 4, 5, 6. Hence, interruption of the migration process is an effective approach for the treatment of glioma. Previous studies showed that α‐mangostin suppressed metastasis in melanoma cells 16, PC‐3 human prostate carcinoma cells 33, MCF‐7 human breast adenocarcinoma cells 34 and pancreatic cancer cells 35. Consistently, our research found that gartanin displayed obvious inhibition of motility in a concentration‐dependent manner *via* wound healing assay and RTCA (Fig. 4). The first step of tumour cells migration is the breakdown of the basement membrane by the activated type IV collagen‐degrading enzymes including MMP‐2 and MMP‐9 36. It has been reported that α‐mangostin suppressed invasion by inhibiting MMP‐2/‐9 33. We also found that gartanin suppressed the proteolytic activities of MMP‐2/‐9 in T98G cells *via* gelatin zymographic assay. To the best of our knowledge, MAPK pathway is one of the most important signalling pathways regulating the synthesis of MMP‐2 and MMP‐9 37, 38. It is reported that activated ERK1/2 or p38 could increase the production of MMPs 39. On the contrary, inhibition of ERK signalling pathway might result in decreased expression of MMP‐2 and MMP‐9 in human breast adenocarcinoma cells 34. To explore the mechanism, we investigated the expression level of p‐ERK, p‐JNK and p‐p38 in T98G cells. Gartanin (10 μM) significantly decreased the phosphorylation of ERK and p38 (Fig. 5). As for p‐ERK, its expression level was increased at the time‐point of 3 hrs (Fig. 5A). Besides anti‐tumour activity, it is also reported that gartanin possessed neuroprotective activity against stress injury 40. In this study, cells were in stress state in the early stage. Gartanin might exert transitorily protective effect against injury through the activation of ERK. However, more studies are needed to reveal the exact mechanism(s). What's more, autophagy had nothing to do with anti‐migration effect of gartanin (Fig. 9). Thus, anti‐migration effect of gartanin could be preliminary explained by down‐regulation of MMP‐2/‐9 as a result of the suppressed MAPK signalling pathway (Fig. 5).

In summary, gartanin, at low micromole, effectively inhibits viability and migration ability of malignant glioma cells mainly by cell cycle arrest and suppression of MMPs. Gartanin, the natural xanthone isolated from the pericarp of mangosteen fruit, might be a promising drug for the treatment of gliomas, alone or combined with other conventional chemotherapy drugs. However, further studies are needed to reveal the exact mechanism(s) and confirm these effects *in vivo*.

## Conflict of interest

The authors declare no conflicts of interest.
